# IL10 Alters Peri-Collateral Macrophage Polarization and Hind-Limb Reperfusion in Mice after Femoral Artery Ligation

**DOI:** 10.3390/ijms21082821

**Published:** 2020-04-17

**Authors:** Alexander M. Götze, Christian Schubert, Georg Jung, Oliver Dörr, Christoph Liebetrau, Christian W. Hamm, Thomas Schmitz-Rixen, Christian Troidl, Kerstin Troidl

**Affiliations:** 1 Department of Trauma-, Hand- and Reconstructive Surgery, Johann-Wolfgang-Goethe-University, 60590 Frankfurt am Main, Germany; 2 Max-Planck Institute for Heart- and Lung-Research, 61231 Bad Nauheim, Germany; christian.schubert@mpi-bn.mpg.de (C.S.); kerstin.troidl@mpi-bn.mpg.de (K.T.); 3 Department of Vascular and Endovascular Surgery, Johann-Wolfgang-Goethe-University, 60590 Frankfurt am Main, Germany; Georg.Jung@kgu.de (G.J.); schmitz-rixen@em.uni-frankfurt.de (T.S.-R.); 4 Department of Cardiology, Justus-Liebig-University, 35392 Gießen, Germany; oliver.doerr@innere.med.uni-giessen.de (O.D.); c.troidl@kerckhoff-fgi.de (C.T.); 5 Department of Cardiology, Kerckhoff Heart and Thorax Center, 61231 Bad Nauheim, Germany; c.liebetrau@kerckhoff-klinik.de (C.L.); c.hamm@kerckhoff-klinik.de (C.W.H.)

**Keywords:** arteriogenesis, collateral artery, macrophages, macrophage polarization, M2 macrophages, IL10

## Abstract

Arteriogenesis is a process by which a pre-existing arterioarterial anastomosis develops into a functional collateral network following an arterial occlusion. Alternatively activated macrophages polarized by IL10 have been described to promote collateral growth. This study investigates the effect of different levels of IL10 on hind-limb reperfusion and the distribution of perivascular macrophage activation types in mice after femoral artery ligation (FAL). IL10 and anti-IL10 were administered before FAL and the arteriogenic response was measured by Laser-Doppler-Imaging perioperatively, after 3, 7, and 14 d. Reperfusion recovery was accelerated when treated with IL10 and impaired with anti-IL10. Furthermore, symptoms of ischemia on ligated hind-limbs had the highest incidence after application of anti-IL10. Perivascular macrophages were immunohistologically phenotyped using CD163 and CD68 in adductor muscle segments. The proportion of alternatively activated macrophages (CD163^+^/CD68^+^) in relation to classically activated macrophages (CD163^−^/CD68^+^) observed was the highest when treated with IL10 and suppressed with anti-IL10. This study underlines the proarteriogenic response with increased levels of IL10 and demonstrates an in-vivo alteration of macrophage activation types in the perivascular bed of growing collaterals.

## 1. Introduction

Arteriogenesis is the process by which a pre-existing arterioarterial anastomosis develops into a functional collateral network following an arterial occlusion. The remodeling processes involved in collateral vessel growth are complex and are dependent on mechanical, cellular and molecular factors [1]. Several authors have demonstrated the pertinence of monocytes and macrophages in enhancing collateral vessel growth [1,2,3]. While there is still debate over whether perivascular macrophages are recruited from circulating monocytes or tissue resident precursors, their proarteriogenic effects, however, are still evident [4,5,6]. Macrophage activation types have become a growing focus in arteriogenesis research, in particular alternatively activated macrophages [7]. Macrophage heterogeneity and plasticity are reflected by their ability to respond to environmental cues giving rise to a spectrum of distinct functional phenotypes or activation states, fulfilling a variety of functions. The extremes of these functional states are commonly defined as M1, M2, or M2-like polarized macrophages [8]. M1 or classically activated macrophages induced by LPS, IFN-γ, and TNF are associated with inflammation and tumor resistance. They differ from M2 macrophages with regard to their arginine metabolism by exhibiting high levels of iNOS and subsequent NO-synthesis, as well as the production of proinflammatory cytokines and chemoattractant proteins. M2/M2-like or alternatively activated macrophages induced by IL4/IL13, immune complexes, agonists of TLR or IL1R, glucocorticoids and IL10, on the other hand, regulate inflammatory responses and promote tissue remodeling, angiogenesis and tumor progression [9]. They are characterized by their arginase/ornithine production, a precursor of cell proliferation, collagen production and ECM remodeling, and the production of anti-inflammatory cytokines [9,10]. While both M1 and M2 macrophages have been shown to contribute to collateral vessel growth, a systemic modulation of known activators of macrophage differentiation demonstrated a determinate proarteriogenic role of M2 macrophages induced by IL10 [11]. This M2 activation phenotype, also referred to as M2c, not only acts as a regulator of immune responses but also as an effector cell of tissue remodeling and repair [9]. It is important to note that the M1/M2 taxonomy of macrophages only represents a limited attempt to categorize the vast variety of functional states observed in vitro. In vivo, this M1/M2 paradigm undermines the complexity of macrophage plasticity and diversity. Functional and phenotypical characteristics of M1 and M2 activation states are not limited to but may instead be shared by more than one macrophage population, allowing them to cater to situational and tissue specific needs. This dogma change has created the need to explore other classifications that more appropriately reflect macrophage behavior in vivo and are subject of current research [12]. Jetten et al. [6] showed that collateral growth was unaffected in mice with a deletion of the IL10 receptor on myeloid cells (IL10R^fl/fl^/LysMCre^+^), arguing that the M2c activation phenotype is not required in arteriogenesis. When treated with exogenously polarized M2c macrophages, however, an improved reperfusion of collateral vessels compared to untreated IL10R^fl/fl^/LysMCre^+^ mice was still observed. As such, this study investigates the in vivo effect of varying levels of IL10 on arteriogenesis as well as the distribution of macrophage activation types around growing collateral vessels.

## 2. Results

### 2.1. Modulation of Blood Concentration Levels of IL10 after Pharmacological Stimulation with IL10 and Anti-IL10

**Figure 1:**
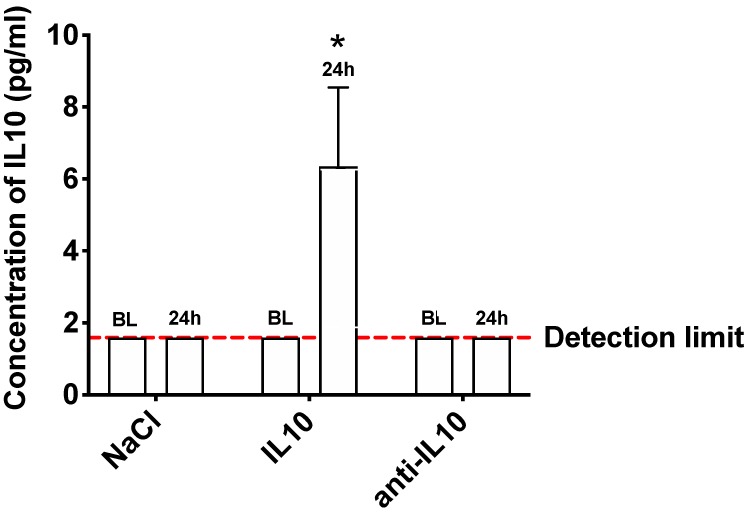
Modulation of blood concentration levels of IL10 after pharmacological stimulation with IL10 and anti-IL10. IL10, anti-IL10, and NaCl were administered via tail vein injection. Blood concentration levels of IL10 were measured before (baseline, BL) and 24 h after pharmacological stimulation. At BL endogenous IL10 levels were below the detection limit of 1.59 pg/mL in all subjects. After an external administration of IL10, blood concentration levels remained significantly increased after 24 h. An effect of anti-IL10 could not be detected, as baseline levels of IL10 remained below the detection limit. * indicates *p* < 0.05; *n* = 3 in each group.

To investigate whether blood concentration levels of endogenous IL10 were affected by an intravenous administration of IL10 or anti-IL10 via tail vein injection, a Mouse Magnetic Luminex Assay was used to determine the blood concentration levels of IL10 prior to (baseline, BL) and 24 h (24 h) after the respective treatments. A control group received NaCl 0.9%. At BL, blood concentration levels of IL10 were below the detection limit of 1.59 pg/mL in all groups. At 24 h, elevated blood concentration levels of IL10 were found only in the IL10 treatment group with 6.35 ± 2.20 pg/mL (*p* < 0.05), indicating that an external administration of IL10 leads to a sustainable change in endogenous blood concentration levels of IL10 for at least 24 h when applied via tail vein injection. Blood concentration levels of IL10 in mice treated with anti-IL10 or NaCl 0.9% remained below the detection limit (Figure 1).

### 2.2. Alteration of Macrophage Polarization in the Perivascular Bed of Growing Collateral Vessels after Pharmacological Stimulation with IL10 and Anti-IL10

**Figure 2:**
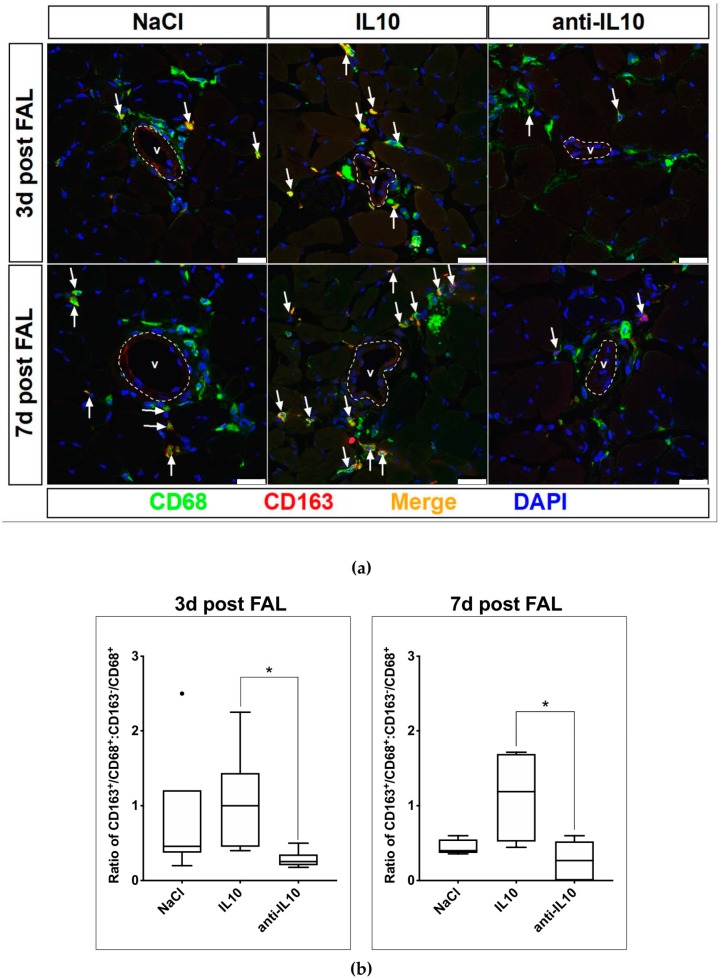
Alteration of macrophage polarization in the perivascular bed of growing collateral vessels after pharmacological stimulation with IL10 and anti-IL10. (**a**) Confocal micrographs of macrophage differentiation subtypes 3 d and 7 d after FAL. Sections of adductor muscles segments containing growing collateral vessels (V) were stained using DAPI and macrophage differentiation markers CD68 and CD163. The ratio of the alternatively (CD163^+^/CD68^+^) activated phenotype, indicated by white arrows, and classically (CD163^−^/CD68^+^) activated phenotype varies with indicated application. Scale bar: 25µm. (**b**) Quantification of macrophage polarization in the perivascular bed of growing collateral vessels after pharmacological stimulation with IL10 and anti-IL10 3 d and 7 d after FAL. The distribution of macrophage subtypes was skewed towards the alternatively activated phenotype after IL10 application. When anti-IL10 was injected, the opposite effect was observed, and the distribution was skewed towards the classically activated phenotype. * indicates *p* < 0.05; 3 d: *n* = 6 in each group; d7: NaCl and IL10 *n* = 4, anti-IL10: *n* = 6.

Adductor muscle samples were harvested 3 days (3 d) and 7 d after FAL to analyze the effect of a treatment with IL10 and anti-IL10 on the polarization of macrophages in the perivascular bed of growing collateral vessels. The samples were sectioned and stained using antibodies targeting known macrophage markers CD68 and CD163 [11,13] (Figure 2a). The two largest collateral vessels of each section were selected and the ratio of macrophages of the alternatively activated phenotype CD163^+^/CD68^+^ to the classically activated phenotype CD163^−^/CD68^+^ per visual field was calculated. Mice treated with NaCl had a median ratio of CD163^+^/CD68^+^ to CD163^−^/CD68^+^ macrophages of 0.46 (IQR: 0.37–1.20) on day 3 (3 d) and 0.40 (IQR: 0.37–0.55) 7 d after FAL. When treated with IL10 the ratio is skewed towards the alternatively activated phenotype on both 3 d and 7 d after FAL with a ratio of 1.00 (IQR: 0.45–1.44) and 1.19 (IQR: 0.52–1.69). Contrariwise, the ratio is skewed towards the classically activated phenotype after application of anti-IL10 on both 3 d and 7 d after FAL with a ratio of 0.25 (IQR: 0.18–0.35) and 0.27 (IQR: 0.00–0.53), differing significantly from that of the IL10 treatment group (*p* < 0.05) (Figure 2b).

### 2.3. Evaluation of Hind-Limb Perfusion Recovery after FAL and Pharmacological Stimulation with IL10 and Anti-IL10

**Figure 3:**
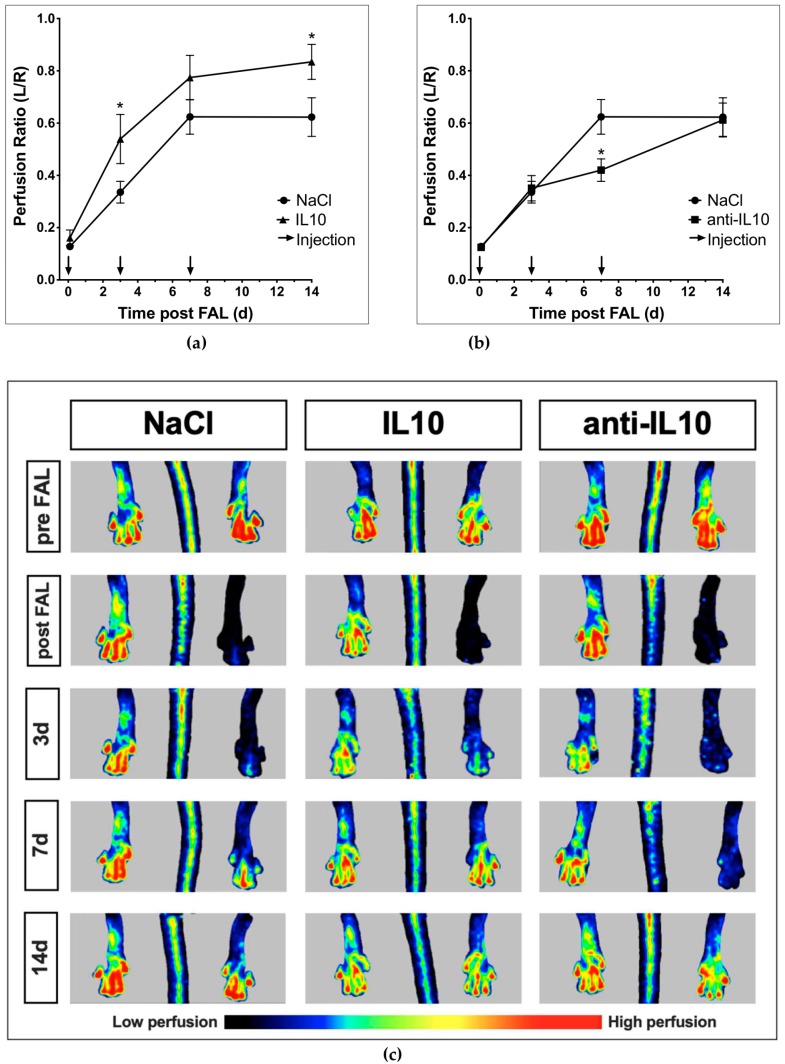
Evaluation of hind-limb perfusion recovery after femoral artery ligation (FAL) and application of IL10 and anti-IL10 (L/R ratio). Hind-limb perfusion was measured prior to and immediately after FAL, on 3 d, 7 d and 14 d. (**a**) IL10 led to a significant acceleration of hind-limb perfusion recovery on 3 d (IL10: 0.54 ± 0.09 vs. NaCl: 0.34 ± 0.04, *p* < 0.05) and 14 d (IL10: 0.83 ± 0.07 vs. NaCl: 0.62 ± 0.07, *p* < 0.05) while (**b**) anti-IL10 led to a brief but significant impairment of hind-limb perfusion recovery on 7 d (anti-IL10: 0.42 ± 0.04 vs. NaCl: 0.62 ± 0.07, *p* < 0.05). (**c**) Representative Laser-Doppler-Images of ligated and non-ligated hind-limbs. *indicates *p* < 0.05; NaCl: *n* = 6, IL10 and anti-IL10: *n* = 5.

To assess the effect of varying blood concentration levels of IL10 on growing collateral vessels IL10 and anti-IL10 were externally applied in mice after FAL. Hind-limb perfusion was assessed using Laser-Doppler-Imaging before and shortly after FAL, on 3 d, 7 d, and 14 d and compared to a control group receiving NaCl. Immediately after FAL an acute reduction of hind-limb perfusion was observed in all groups (NaCl: 0.13 ± 0.01, IL10: 0.16 ± 0.03, anti-IL10: 0.13 ± 0.01). Hind-limb reperfusion in the NaCl (*n* = 6) group showed an adequate increase to 0.34 ± 0.04, 0.62 ± 0.07, and 0.62 ± 0.07 on 3 d, 7 d, and 14 d respectively. Elevated blood concentration levels of IL10 led to a significantly higher hind-limb perfusion on 3 d and 14 d (*n* = 5, 3 d: 0.54 ± 0.09, *p* < 0.05, 14 d: 0.83 ± 0.7, *p* < 0.05). Although hind-limb perfusion on 7 d was elevated compared to the control group, the difference was not significant (0.77 ± 0.09, *p* = 0.07) (Figure 3a). Contrariwise, application of anti-IL10 showed a significant impairment of hind-limb perfusion on 7 d (*n* = 5, 0.42 ± 0.4, *p* < 0.05). On 3 d and 14 d, however, no significant difference was observed (3 d: 0.35 ± 0.05, *p* = 0.86, 14d: 0.61 ± 0.07, *p* = 0.89) (Figure 3b).

### 2.4. Macroscopic Observations on Ligated Hind-Limbs after FAL and Application of IL10 and Anti-IL10

**Figure 4:**
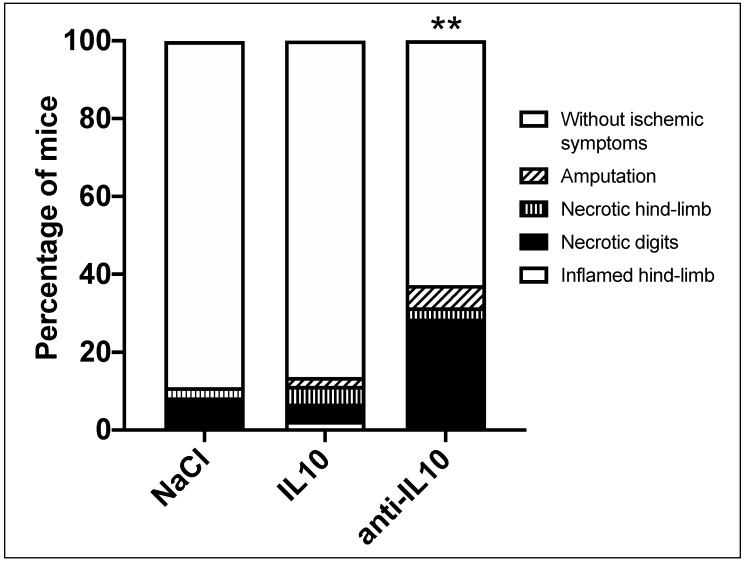
After FAL and pharmacological stimulation with IL10 or anti-IL10 the onset of critical ischemic events (CIE) were recorded. The highest incidence of CIE was observed after application of anti-IL10 (anti-IL10: 37.1% vs. NaCl: 11.1% *p* < 0.01, IL10: 13.6%). **indicates *p* < 0.01; NaCl: *n* = 36, IL10: *n* = 44, anti-IL10: *n* = 35

Ligated hind-limbs were inspected prior to FAL, 3 d and 7 d after FAL. Macroscopic observations associated with an acute ischemia of the affected hind-limb, termed critical ischemic events (CIE), were recorded after FAL and treatment with IL10 and anti-IL10. CIE were categorized as follows: inflamed hind-limb, necrotic digits, necrotic hind-limb and amputation. In total (*n* = 115) CIE occurred in 20%. Necrotic digits were observed most frequently in 65%, followed by a necrotic hind-limb in 17%, an amputation in 13% and an inflamed hind-limb in 4% of CIE. The highest incidence of CIE was observed after application of anti-IL10 (anti-IL10: 37.1% vs. NaCl: 11.1% *p* < 0.01). The incidence of CIE when treated with IL10 was similar to the control group (IL10: 13.6% vs. NaCl: 11.1% *p* = 0.73) (Figure 4).

## 3. Discussion

In this study we have shown that modulation of IL10, in particular elevated levels of circulating IL10, alters collateral reperfusion after femoral artery ligation (FAL) as well as the distribution of macrophage activation types in the perivascular bed of growing collaterals. Although, the inhibitory effect of an IL10 antibody on circulating IL10 blood levels could not be directly detected due to technical limitations, contrary to elevated levels of circulating IL10, the opposite pertaining to both collateral reperfusion and the distribution of macrophage activation types was observed. The remodeling processes in arteriogenesis involve a controlled destruction of vessel components, activation of endothelial and smooth muscle cell de-differentiation, proliferation and migration, and adventitial restructuring in which monocytes/macrophages were shown to be key orchestrators [1,14]. These seemingly disparate tasks mediated largely by a singular cell type are explained by their heterogeneity and plasticity in response to environmental and situational needs. Previous findings have demonstrated that M1 and M2 macrophages form around growing collateral vessels in a distinct temporal and spatial pattern suggesting a collaborative mechanism of action in collateral artery growth [11]. The M1 phenotype, found proximate to the vessel lumen, is associated with the production of the pro-inflammatory cytokines IL1 and IL6, both described to have autocrine growth effects on vascular smooth muscle cells (VSMC) [15]. Other arteriogenic stimulants expressed by the M1 phenotype include NOS, TNF and MCP-1 [1,2,15,16]. M2 macrophages, on the other hand, are found distal to the vessel lumen [11] and have been ascribed a more prominent role in mediating the growth processes. Takeda et al. [7] showed that improved collateral perfusion after FAL was due to an expansion of the M2 phenotype among tissue-resident macrophages in Phd2 haplodeficient (Phd2^+/-^) mice and that soluble factors secreted by Phd2^+/-^ macrophages in vitro led to increased smooth muscle cell (SMC) proliferation and migration. IL10 is a known activator of the M2c phenotype and we have shown that increased levels of IL10 also led to an improved collateral reperfusion after FAL. Furthermore, an immunohistochemical analysis using the established macrophage polarization marker CD163 [17,18], revealed that perivascular macrophages were proportionally skewed towards the M2 phenotype. The supposed proarteriogenic effects of M2 macrophages induced by IL10 are further supported by the observation that the application of an IL10 antibody led to a transient impairment of collateral reperfusion and was accompanied by an increased onset of ischemic symptoms on ligated hind limbs. Also, the distribution of perivascular macrophages was conversely skewed towards the M1 phenotype. These findings do not undermine the role of M1 macrophages in arteriogenesis. The delayed and transient dip in reperfusion recovery we observed after the application of an IL10 antibody may support the hypothesis, that adluminally located M1 macrophages contribute to collateral vessel growth particularly in early phases through the recruitment of circulating monocytes and expression of arteriogenic relevant cytokines and proteins (IL1, IL6, TNF, NOS, MCP-1). They do, however, highlight the potential role played by M2 macrophages with regard to VSMC differentiation and adventitial restructuring to accommodate the growing collateral vessel. M2 macrophages induced by IL10 secrete high levels of TGFb1, known to stimulate VSMC differentiation and extracellular matrix (ECM) deposition [15,19,20]. They also produce high levels of MMP9 [21], which along with MMP2 was significantly increased in the adventitia of growing coronary collateral vessels [22]. This supports the hypothesis that IL10 induced M2 macrophages may play an active role in the augmentation of adventitial ECM proteolysis and remodeling, thus, facilitating arteriogenic growth. Our findings suggest that varying levels of IL10 in vivo influence collateral reperfusion, which may be explained by a shift in the distribution of macrophages towards the M2 phenotype at the site of collateral growth. From a clinical standpoint, this presents itself as a new therapeutic approach to promote collateral vessel growth in patients suffering from peripheral artery disease. The expression of CD163 on M2 macrophages, however, also poses concerns regarding their use in revascularization therapy. CD163^+^ macrophages in human atherosclerotic lesions were found to increase plaque instability by promoting angiogenesis within areas of intraplaque hemorrhage, in itself thought to be a result of plaque neovascularization and increased microvessel permeability, resulting in a vicious cycle [23]. Seen as a whole, these observations underline the diversity of macrophage functions and activation states with regard to tissue specific cues and needs. A mere distinction between classically activated M1 and alternatively activated M2 macrophages limited by CD163 as an M2 marker alone will not suffice to fully describe the roles played by the macrophage activation states we observed around growing collateral vessels. Further studies utilizing other macrophage markers will be required to elucidate the underlying mechanism involved in our findings. While these remain hypothetical, the results presented in our study shed light on new therapeutic strategies in promoting collateral vessels growth.

## 4. Materials and Methods 

### 4.1. Animal Models

Animal handling and all experimental procedures carried out were in full compliance with the Directive 2010/63/EU of the European Parliament on protection of animals used for scientific purposes. Approval was given by the responsible local authority, the hessian governmental council for animal protection and handling (permit reference numbers V54-19c20/15-B2/1152, permit date: 23.05.2017). Throughout this study all mice had access to water and food ad libitum.

### 4.2. Mouse Model of Hind-Limb Ischemia

To evaluate collateral vessel growth perfusion recovery was measured after femoral artery ligation (FAL) using the model described in [24]. For all experiments 10-14 weeks old male C57BL/6 mice from our own breeding program were used with an approximate bodyweight of 30 g. Prior to each experiment all mice were inspected to ensure a healthy state. Anesthesia was performed by intraperitoneal injection using ketamin hydrochloride (120mg/kg bodyweight) and xylazine hydrochloride (16 mg/kg bodyweight). Pre- and post-operative analgesia was performed by subcutaneous injection with buprenorphine (0.1 mg/kg bodyweight). FAL was carried out on the left hind-limb by ligating the femoral artery immediately distal to the origin of the deep femoral branch to redirect blood flow to the collateral arteries. After termination of experiments the mice were euthanized by an anesthetic overdose using ketamin hydrochloride (180mg/kg bodyweight) and xylazine hydrochloride (16 mg/kg bodyweight) followed by exsanguination.

### 4.3. Pharmacological Stimulation

FAL was performed on C57BL/6 mice. Mice were randomly allocated to each group receiving either recombinant murine interleukin 10 (IL10) (20 µg/kg bodyweight) or purified anti-mouse IL10 antibody (anti-IL10) (0.5 µg/kg bodyweight) diluted in sodium chloride solution (NaCl 0.9%) immediately after FAL, on day 3 and 7 after FAL. The control group received NaCl 0.9%. The application was carried out via intravenous injection into the tail vein. IL10 was purchased from PeproTech (Hamburg, Germany). Anti-IL10 was purchased from BioLegend (Koblenz, Germany).

### 4.4. Measurement of Blood Concentration Levels of IL10

Retro orbital blood samples were obtained from mice before and 24 h after pharmacological stimulation with IL10 and anti-IL10 as described above. The control group received NaCl 0.9%. Anesthesia war provided as described above. A Mouse Magnetic Luminex Assay (Thermo Fisher Scientific, Waltham, MA, USA) was used to measure the concentration of endogenous IL10 in the blood samples at baseline and 24 h after pharmacological stimulation.

### 4.5. Hind-limb Perfusion Measurement after Pharmacological Stimulation and FAL

Hind-limb perfusion was assessed and quantified via erythrocyte motion detection through Laser-Doppler-Imaging using a PeriScan PIM3 System (Perimed Instruments, Järfällä, Sweden, Software: LDPIwin for PIM3 3.1.3) before and immediately after FAL, on day 3, 7 and 14 after FAL. For each measurement mice were positioned on a heating plate at 37 °C for 3min prior to and during each measurement to ensure standardized conditions at a distance of 10 cm and a pixel resolution of 256 × 256. A 2 cm × 3 cm area including both feet was scanned and a region of interest (ROI) of approximately 80 mm^2^ containing each foot was defined. Mean perfusion (arbitrary units) was used to calculate hind-limb perfusion and expressed as the ligated limb to non-ligated limb ratio as described in [25]. Follow-up measurements were performed under anesthesia as described above.

### 4.6. Immunohistochemistry

Mice were perfused with 10 mL vasodilation buffer (100 µg adenosine, 1 µg sodium nitroprusside, 0.05% BSA in PBS, pH 7.4) followed by 10 mL 4% PFA post mortem. Mm. adductores of ligated hind-limbs were harvested on day 3 after FAL and cryosectioned with a thickness of 10 µm. Immunostaining was performed using the following antibodies: RAT ANTI MOUSE CD68: Alexa Fluor 488 Antibody (AbD Serotec, Düsseldorf, Germany), CD163 (M-96) Antibody (Santa Cruz Biotechnology Inc., Dallas, TX, USA), Donkey anti-Rabbit IgG: Alexa Fluor 546 (Thermo Fisher Scientific, Waltham, MA, USA), DAPI. The two largest collateral vessels were selected. Immunopositive cells were classified as classically activated macrophages (CD68^+^/CD163^−^) or alternatively activated macrophages (CD68^+^/CD163^+^). Confocal imaging was carried out using a Leica SP5 (Wetzlar, Germany). Acquired images were processed with ImageJ Software (National Institutes of Health, Maryland, MD, USA) for further analysis.

### 4.7. Data Analysis

Statistical analysis was performed using Prism (GraphPad Software, San Diego, USA). Data was tested for normality using a D’Agostino-Pearson omnibus normality test. Parametric data is reported as mean ± standard error of mean. Non-parametric data is reported as median (IQR). Unpaired and parametric samples were analyzed using a Student’s t-test. Unpaired and non-parametric samples were analyzed using a Mann–Whitney-U test. When more than two groups were compared at different time points, a two-way ANOVA was used for parametric samples followed by a Holm–Sidak’s multiple comparison test. For comparison of more than 2 groups with non-parametric samples a Kruskal–Wallis test was used followed by a Dunn’s multiple comparison test. Proportions were compared using a Chi-square test. Values of *p* < 0.05 were considered statistically significant.

## Abbreviations

FAL	Femoral artery ligation
CIE	Critical ischemic events
VSMC	Vascular smooth muscle cells
SMC	Smooth muscle cells
ECM	Extracellular matrix
